# Supplementary material for: Reprocessing filtering facepiece respirators in primary care using medical autoclave: prospective, bench-to-bedside, single-centre study
Source: BMJ Open. 2020 Aug 4;10(8):e039454. doi: 10.1136/bmjopen-2020-039454 (PMC7406112; doi:10.1136/bmjopen-2020-039454)
Supplement: Author's manuscript [file bmjopen-2020-039454.draft_revisions.pdf]

BMJ Open

BMJ Open is committed to open peer review. As part of this commitment we make the peer review history of every article we publish publicly available.

When an article is published we post the peer reviewers' comments and the authors' responses online. We also post the versions of the paper that were used during peer review. These are the versions that the peer review comments apply to.

The versions of the paper that follow are the versions that were submitted during the peer review process. They are not the versions of record or the final published versions. They should not be cited or distributed as the published version of this manuscript.

BMJ Open is an open access journal and the full, final, typeset and author-corrected version of record of the manuscript is available on our site with no access controls, subscription charges or pay-per-view fees (http://bmjopen.bmj.com).

If you have any questions on BMJ Open's open peer review process please email info.bmjopen@bmj.com

BMJ Open

Decontamination of filtering facepiece respirators in primary care using medical autoclave

Journal:	*BMJ Open*
Manuscript ID	bmjopen-2020-039454
Article Type:	Original research
Date Submitted by the Author:	16-Apr-2020

Complete List of Authors:	Harskamp, Ralf; Amsterdam UMC - Locatie AMC, General Practice<br>van Straten, Bart; Delft University of Technology; Van Straten Medical<br>Bouman, Jonathan; Amsterdam UMC - Locatie AMC<br>van Maltha - van Santvoort, Bernadette; Holendrecht Medical Center<br>van den Dobbelsteen, John; Delft University of Technology<br>van der Sijp, Joost; Medical Centre Haaglanden; GreenCycl<br>Horeman, Tim; Delft University of Technology

Keywords:	PRIMARY CARE, Respiratory infections < THORACIC MEDICINE, Health & safety < HEALTH SERVICES ADMINISTRATION & MANAGEMENT

SCHOLARONE™
Manuscripts

I, the Submitting Author has the right to grant and does grant on behalf of all authors of the Work (as defined in the below author licence), an exclusive licence and/or a non-exclusive licence for contributions from authors who are: i) UK Crown employees; ii) where BMJ has agreed a CC-BY licence shall apply, and/or iii) in accordance with the terms applicable for US Federal Government officers or employees acting as part of their official duties; on a worldwide, perpetual, irrevocable, royalty-free basis to BMJ Publishing Group Ltd ("BMJ") its licensees and where the relevant Journal is co-owned by BMJ to the co-owners of the Journal, to publish the Work in this journal and any other BMJ products and to exploit all rights, as set out in our licence.

The Submitting Author accepts and understands that any supply made under these terms is made by BMJ to the Submitting Author unless you are acting as an employee on behalf of your employer or a postgraduate student of an affiliated institution which is paying any applicable article publishing charge ("APC") for Open Access articles. Where the Submitting Author wishes to make the Work available on an Open Access basis (and intends to pay the relevant APC), the terms of reuse of such Open Access shall be governed by a Creative Commons licence – details of these licences and which Creative Commons licence will apply to this Work are set out in our licence referred to above.

Other than as permitted in any relevant BMJ Author's Self Archiving Policies, I confirm this Work has not been accepted for publication elsewhere, is not being considered for publication elsewhere and does not duplicate material already published. I confirm all authors consent to publication of this Work and authorise the granting of this licence.

For peer review only

Decontamination of filtering facepiece respirators in primary care using medical autoclave

Ralf E. Harskamp [1,2]*, Bart van Straten [3,4]*, Jonathan Bouman [1,2], Bernadette van Maltha – van Santvoort [2], John J. van den Dobbelsteen [3], Joost R.M. van der Sijp [5,6], Tim Horeman [3]

[1] Department of General Practice, Amsterdam UMC – location AMC, Amsterdam, the Netherlands
[2] Holendrecht Medical Center, Amsterdam, the Netherlands
[3] Department of BioMedical Engineering, Faculty of Engineering, Delft University of Technology, Delft , the Netherlands
[4] Van Straten Medical, De Meern, the Netherlands
[5] GreenCycl, De Meern, the Netherlands
[6] Haaglanden Medical Center, The Hague, the Netherlands

* both authors contributed equally to this study

Author for correspondence:
Name: Dr Ralf E. Harskamp, MD, PhD
Role/title: Assistant Professor
Department: Department of General Practice, Division of Primary Care, Public Health and Methodology
Institution: Amsterdam University Medical Centers – Location Academic Medical Center
Address: Room: J2-126, Meibergdreef 9, 1105 AZ, Postbox 22660, 1100 DD Amsterdam, The Netherlands.
Phone: +31(0)20-566.7457
Fax: +31(0)20-566.9186
E-mail for correspondence: r.e.harskamp@gmail.com
Alternative e-mail address : r.e.harskamp@amsterdamumc.nl

For peer review only

Abstract:

Objective
There are widespread shortages of personal protective equipment as a result of the coronavirus disease 2019 (COVID-19) pandemic. Reprocessing filtering facepiece respirators may provide an alternative solution in keeping health care professionals safe.

Design: prospective, bench-to-bedside

Setting: A primary care-based study using filtering facepiece particles (FFP) type 2 respirators without exhalation valve (3M Aura 1862+, Maco Pharma ZZM002), FFP2 respirators with valve (3M Aura 9322+ and San Huei 2920V), and valved FFP type 3 respirators (Safe Worker 1016).

Interventions: All masks were reprocessed using a medical autoclave (34-minute total cycle time of steam sterilization, with 17 minutes at 121°C) and subsequently tested up to 3 times whether these decontaminated respirators retained their integrity (seal check, pressure drop) and ability to filter small particles (0.3-5.0µm) in the laboratory using a particle penetration test.

Results
We tested 32 respirators, and 63 samples for filter capacity. All 27 FFP-2 respirators retained their shape, whereas half of the sterilized FFP-3 respirators (Safe Worker 1116) showed deformities and failed the seal check. The filtering capacity of the 3M Aura 1862 was best retained after 1, 2, and 3 sterilization cycles (0.3µm: 99.3±0.3% (new) versus 97.0±1.3, 94.2±1.3% or 94.4±1.6, $p<0.001$). Of the other FFP-2 respirators, the San Huei 2920V had 95.5±0.7% at baseline versus 92.3±1.7% versus 90.0±0.7 after one- and two-time sterilization, respectively ($p<0.001$). The tested FFP-3 respirator (Safe Worker 1016) had a filter capacity of 96.5±0.7% at baseline and 60.3±5.7% after one-time sterilization ($p<0.001$). Breathing and pressure resistance tests indicated no relevant pressure changes between respirators that were used once, twice or thrice.

Conclusion
This study shows that selected FFP2-type respirators may be reprocessed for use in primary care, as the tested masks retain their shape, ability to retain particles and breathing comfort after decontamination using a medical autoclave.

For peer review only - http://bmjopen.bmj.com/site/about/guidelines.xhtml

Strengths and limitations of this study

- Pragmatic use of autoclave to sterilize and reuse filter facepiece respirators

- Combining clinical and laboratory findings to evaluate the safety in terms of shape, ability to retain particles and breathing comfort

- The study is limited in sample size and restricted to selected FFP-2 and FFP-3 respirators

- The study is a first of its kind in primary care settings and thus unvalidated

- The study does not provide "hard" clinical evidence in terms of a randomized trial (i.e. reprocessed mask versus usual care)

For peer review only

For peer review only - http://bmjopen.bmj.com/site/about/guidelines.xhtml

Introduction

General practitioners (GP) are often the first to evaluate patients with (suspected) coronavirus disease 2019 (COVID-19). This is particularly true in countries where GPs have a gatekeeping role. Given the risk of person-to-person spread this necessitates the need to wear personal protective equipment. [1,2] Unfortunately, most health care facilities are running dangerously low on this equipment. [1,2] In the United States, these critical shortages have resulted in downgrading from respirators to surgical masks and now even resort to home-made cloth-masks. [1] Access to adequate supplies is crucial to preventing transmission of pathogens, especially in resource-limited settings. [3] Reports across several countries found that healthcare workers are more at risk of catching SARS-CoV2 as well as at higher risk of severe COVID-19, possibly due to exposure to higher viral load. [4] The outbreak of COVID-19 in Italy showed that inadequate access to protective equipment is one of the reasons why healthcare workers, and particularly GPs, experienced high rates of infection. [2] Aside from the direct health effects, absenteeism from illness may also negatively affect the health system's capacity to adequately respond to the COVID-19 pandemic. Moreover, it makes healthcare workers feel unsafe and unprotected, which undermines morale as shown in a report on England's National Health Service health workers experiences. [5]

One of the possible (short-term pragmatic) solutions could be the reuse of equipment, and in particular that of respirators. To reuse a mask or respirator, it should be sterilized first. The method applied should: 1) kill the SARS-CoV-2 virus (diminish the viral load); 2) keep the mask's protective properties (largely) intact, in terms of filter and fit. In primary care, the medical autoclave is normally used to sterilize surgical instruments. The process of pressurized moist heat destroys microorganisms by the irreversible coagulation and denaturation of enzymes and structural proteins, and has been shown to be effective in respirators contaminated with other viruses, such as H1N1 influenza virus. [2, 6, 7] However, the question is whether the respirator's protective properties in terms of filter function and fit will remain intact when exposing the respirator to steam. We therefore set out to study whether the process of steam sterilization negatively affects the protective properties of commonly used respirators which are designed to protect the wearer against the inhalation of both droplets and particles suspended in the air.

Methods

We reported our findings according to the Better reporting of interventions: template for intervention description and replication (TIDieR) checklist and guide, as well as the general principles of reporting a study using the directions provided by the journal. [8]

Study design and setting

The study involved the evaluation of available filtering respirators used to evaluate suspected COVID-19 patients in the Holendrecht Medical Center, in Amsterdam, the Netherlands. For high-risk patients, the center provides GPs with filtering facepiece particles (FFP) type 2 or type 3 for personal protection. For the current study, worn respirators were used for reprocessing using a medical autoclave. After the autoclave procedure the respirators were visually inspected for deformity by two clinical investigators, followed by a seal check. The masks were subsequently marked and sent by courier to the GreenCycl testing laboratory in Utrecht, the Netherlands. At this

For peer review only - http://bmjopen.bmj.com/site/about/guidelines.xhtml

facility, the sterilized respirators were tested by two laboratory scientists for their filter capacity. For comparison, the results ware compared with the filter capacity of unused, brand-new respirators that were used as a reference. Moreover, a pressure drop test was performed to evaluate whether the breathing resistance altered by the process of sterilization.

Sterilization process

The masks were sterilized for multiple cycles using a cylindrical chamber tabletop autoclave (Kronos S18, release: E.5.47a, Newmed, Quattro Castella, Italy). This type of vacuum autoclave is typically designed for general practitioner and dental practices and has pre-programmed cycles. The size as well as the cycle times differ from autoclaves typically used in hospitals, which are larger and have longer cycle times, however with comparable peak times regarding sterilization. The Kronos S18 autoclave holds a capacity of 18L or 4 respirators. The autoclave has specific programs for "solid made of rubber and delicate solids", which includes respirators. The sterilization program we used involved a 34-minute cycle, of which the first 12 minutes of the cycle involved preheating, followed by 17 minutes steam sterilization at a temperature of 121 degrees of Celsius, and finished with a 5-minute drying process.

Visual inspection, breathing resistance, and user seal check

After sterilization the respirators were checked for visual deformities of the mask as well as the elastic straps. Subsequently, the respirators were put on to evaluate whether breathing felt normal, followed by the performance of a user seal check. A negative pressure user seal check was used for all respirators in which the clinical investigator inhaled sharply while blocking the paths for air to enter the facepiece. A successful check is when the facepiece collapsed slightly under the negative pressure that was created with this maneuver. For respirators without an exhalation valve the investigator also performed a positive pressure check by exhaling gently while blocking the paths for air to exit the facepiece. A successful check is when the facepiece was slightly pressurized before increased pressure causes outward leakage. [9]

Particle penetration test

At the testing laboratory two independent researchers from the Delft University of Technology tested the masks using a dry particle penetration test setup (***Figure 1A***). [10] The equipment involved a SOLAIR 3100 particle counter (Lighthouse Worldwide Solutions Inc, Fremont, CA). The particles are counted within the machine via a tube that is connected to a particle chamber to which the respirator is secured. The transparent lid presses the mask such that it prevents material buckling and creates an airtight seal that only allows air to pass through the material. Before each measurement, a benchmark test is conducted with 28 Liters of surrounding air that is sucked through the particle chamber into the particle counter. During this measurement no mask is installed. During the test measurement, a mask is installed on the particle chamber. Therefore, the 28 liter of surrounding air is sucked through the filter material of the mask and the remaining particles are counted in the categories of 0.3, 0.5, 1.0 and 5.0 microns. The measurements are compared and the filtering capacity derived based on the difference in the readings compared to the benchmark test. A lower number of particles counted after filtering in relation to the benchmark test would indicate better filtering performance. [11] The system setup is more conservative than the NEN-149 standard, which means the resulting filter capacity percentages cannot be translated

For peer review only - http://bmjopen.bmj.com/site/about/guidelines.xhtml

directly to the known FFP1, 2 and 3 standards. However, as the filter capacity of a new mask is known, the measurement results do show the remaining filter capacity and therefore indicates whether a mask type deteriorates after steam sterilization.

Flow resistance

The setup was expanded with an additional pressure sensor (SDP 816-500Pa Sensirion #1230319) and flow adjustment valve in order to investigate whether the pressure delta over the mask material changed after sterilization cycles. (***Figure 1B***) The Sensirion pressure sensor was connected with a T-piece between the particle chamber and Lighthouse 3100. An additional valve was used to adjust the input pressure within range of the sensor. A LabJack T7 analogue input device was used to convert the output from the pressure sensor to an output voltage of 0 to 5 Volt. An output value of 5V was representing 500Pa and set as 100% of input pressure. the atmospheric input pressure of 2.42 Volt was defined as 0% output. Measurements were conducted with a constant air speed of 20.7 meter per seconds at the opening of the particle chamber.

Outcomes of interest

The outcomes of interest involved: 1) signs of deformity of the respirator, which was performed by visual inspection; and 2) the percentage of filtered particles with a diameter of 0.3 µm. This diameter is clinically relevant, given that to meet the FFP-2 standards a mask should filter 94% of all 0.3 µm particles, whereas 99% of these particles should be filtered to meet the FFP-3 standard.

Statistical analysis

The study involved descriptive analyses, with numbers and percentages, and comparisons were performed using an alpha of 0.05 for statistical significance. The findings of the filter tests are visually displayed in Box plots, and presented as mean and standard deviation. We used JASP statistical software (version 0.10.2, University of Amsterdam, the Netherlands).

Patient and Public Involvement:

Patients and/or the public were not involved in the design, or conduct, or reporting, or dissemination plans of our research.

Results

We obtained 32 respirators, of which 27 were used during consultation or high-risk home visits of COVID-19 suspected patients at the Holendrecht Medical Center in March/April 2020. The facemasks were FFP-2 respirators (3M Aura 1862+, Maco Pharma ZZM002), FFP2 respirators with exhalation valve (3M Aura 9322+ and San Huei 2920V), or FFP3 respirators (Safe Worker 1016). The 27 used respirators (including 4 FFP3 respirators) underwent sterilization, with the remaining 5 serving as a reference (as they did not to undergo sterilization).

Visual inspection, breathing resistance, and user seal check

After the sterilization process all FFP-2 respirators retained their shape and were without visible damage. When fitting, the elastic bands of all masks still functioned normally, with no difference from non-sterilized masks in terms of breathing resistance. The seal checks also did not reveal significant air leakage suggesting poor fit. However,

For peer review only - http://bmjopen.bmj.com/site/about/guidelines.xhtml

unlike the FFP-2 respirators, two out of the four FFP3 respirators (50%) showed signs of deformation, with a crumbled appearance, and failed seal check test.

Filter capacity of sterilized respirators
For the particle penetration test, a total of 63 samples were tested from 32 respirators. The results of the filter capacity for 0.3 microns are illustrated in ***Figure 2*** and for larger particles are displayed in ***Table 1.*** Of the tested FFP-2 respirators we found that the 3M Aura 1862+ remained close to its original filtering capacity after one-, two-time, and three-time sterilization (0.3µm: 99.3±0.3% versus 97.0±1.3, 94.2±1.3% or 94.4±1.6, respectively, p<0.001). The 3M Aura 9322+ (with valve) had a filter capacity of 96.8±0.2% without sterilization versus 91.0±1.4% and 77.5±2.1% after one- or two-time sterilization (p<0.001). The Maco Pharma ZZM002 FFP-2 mask did not have a reference mask, but after one- and two-time sterilization the filter capacities were 89.3±3.9% and 86.6±2.6%, respectively. The San Huei 2920V respirator had 95.5±0.7% at baseline versus 92.3±1.7% versus 90.0±0.7 after one- and two-time sterilization (p<0.001). Finally, the tested FFP-3 respirator (Safe Worker 1016) had a filter capacity of 96.5±0.7% at baseline and 60.3±5.7% after one-time sterilization (p<0.001).

Flow resistance
For the breathing resistance test we tested 6 FFP-2 respirators (3M Aura 1862+): two were used once and reprocessed, two were used twice and reprocessed after each use and two were used for 3 times and reprocessed after each use. The average pressure did not increase with the number of reuses (35.6±0.3%, 35.4±0.0%, 36.7±0.3%, respectively)

Discussion
The COVID-19 pandemic has caused major shortages of PPE, including protective respirators. While production has increased, shortages are so high that reprocessing of used respirators and respirators is probably one of the only viable short-term solutions. In primary care, a tabletop autoclave would be a pragmatic choice, as the device is readily available in practices for sterilization of surgical and gynecological instruments. In this study we found that steam sterilization at 121 degrees Celsius may provide a viable option for selected respirators, but it also sheds a light on the variability in the protective properties of the various available respirators and respirators. Of the tested respirators, the filter capacity of the 3M Aura 1862+ respirator fared best with a consistently high filter capacity for the 0.3 µm particle size category and above after multiple cycles of steam sterilization. Moreover, there are no indications that the respirator becomes harder to breathe through and thus more uncomfortable to wear.

<u>*Findings in relationship to FFP-2 and FFP-3 standards*</u>
The particle chamber used in this study appears to be more stringent (more sensitive) that the NEN-149 criteria that are used for FFP-2 and FFP-3 norms. We performed a cross-check with 4 KN95 respirators which showed that measurements of 67% and 82% particle retention at 0.3 and 0.5 microns on average, using our setup still resulted in approval for use according to the FFP-2 norm when measured according to the NEN-149, based on a continuous flow setup (Kalibra, Delft, the Netherlands). Therefore, apart from the Safe Worker 1016, all other mask types will likely still comply with NEN-149 FFP-2 threshold values.

For peer review only

Study limitations

Our study involved a pragmatic study with a limited sample size of respirators and respirators available in our practice. Our study did not involve testing of surgical masks or FFP-1 masks, and we do not know whether reprocessing of these materials would still provide adequate protection based on their respective standards. Furthermore, we did not perform a laboratory-based Fit test. Also, we presumed that solid particles of 0.3-0.5 microns are of relevance and behave similar as droplets that normally carry viruses from one person to another. Moreover, we do not know exactly at what particle size viral transmission is still possible. Finally, this study did not test the efficacy of reprocessed respirators in a clinical trial setting, as such we do not know the “real world” safety of reprocessed respirators.

Prior studies

In our primary care medical center, we have empirically experimented with decontamination of FFP-2 and FFP-3 respirators and respirators using steam sterilization using the medical autoclave. We found that when using steam sterilization at higher temperatures (132-140 centigrade), visual deformities occurred on the respirators, particularly those with a plastic respirator valve. A recent analysis of the Dutch Centers of Disease Control (RIVM) also found that steam sterilization at higher temperatures deformed the masks [12]. To our knowledge there is only one prior study that assessed the impact of steam sterilization on the filter capacity of facepiece respirators, which was a study by Lin and colleagues in 2017. [13] In this study, the authors found that one of the decontamination processes that appeared effective for N95 respirators was the medical autoclave, in which they exposed the respirators to saturated steam at 121 degrees Celsius for 15 minutes. They found that filter quality (≥95%) of the masks remained intact using a range of particles. These findings are comparable to those we present in this paper.

Implications for practice

For COVID-19 like for other viruses, transmission can occur via droplets or aerosols. [1-4] Thus personal protection is warranted to avoid catching COVID-19. Currently there is no evidence on which type of face mask offers best protection for COVID-19. Prior studies with influenza viral particles showed that FFP2 respirators may provide better protection that surgical masks, when used appropriately. [14] In this COVID-19 pandemic, it is thought that the use of surgical masks may be sufficient for consultations with only limited person-to-person exposure. However, it is much less certain whether surgical masks will provide adequate protection during longer consultations or back-to-back consultations with patients with suspected COVID-19 in a closed consultation room. [15] In these instances, respirators may be preferable. Given, the limited availability, reusing FFP-2 type respirators may provide a second-best alternative that can be readily performed in primary care and other low-resource settings using a table-top medical autoclave, as described in this study.

Conclusion

This study shows that selected FFP2 respirators may be reprocessed for use in primary care, as the respirators retain their shape, ability to retain particles and breathing comfort after decontamination using a medical autoclave. However, future studies are warranted to confirm our findings.

For peer review only - http://bmjopen.bmj.com/site/about/guidelines.xhtml

References

[1] https://www.cdc.gov/coronavirus/2019-nCoV/hcp/index.html

[2] Ranney ML, Griffeth V, Jha AK. Critical supply shortages - the need for ventilators and personal protective equipment during the Covid-19 pandemic. N Engl J Med 2020; March 25th, https://www.nejm.org/doi/full/10.1056/NEJMp2006141

[3] Honda H, Iwata K. Personal protective equipment and improving compliance among healthcare workers in high-risk settings. Curr Opin Infect Dis 2016;29:400-6

[4] Heneghan C, Brassey J, Jefferson T. SARS-CoV-2 viral load and the severity of COVID-19. https://www.cebm.net/covid-19/sars-cov-2-viral-load-and-the-severity-of-covid-19/

[5] Horton R. Offline: COVID-19 and the NHS – "a national scandal". The Lancet 2020;395:P01229.

[6] Rutala WA, Weber DJ. Sterilization in healthcare facilities, 2008. Update: May 2019. https://www.cdc.gov/infectioncontrol/guidelines/disinfection/

[7] Heimbuch BK, Wallace WH, Kinney K, Lumey AE, Wu C-Y, Woo M-H, Wander JD. A pandemic influence preparedness study: use of energetic methods to decontaminate filtering facepiece respirators contaminated with H1N1 aerosols and droplets. Am J Infect Control 2011;39:e1-9.

[8] Hoffmann TC, Glasziou PP, Milne R, et al. Better reporting of interventions: template for intervention description and replication (TIDieR) checklist and guide. BMJ 2014;348:g1687

[9] NIOSH. Filtering out Confusion: Frequently Asked Questions about Respiratory Protection, User Seal Check. By Krah J., Shamblin M., and Shaffer R. Pittsburgh, PA: U.S. Department of Health and Human Services, Centers for Disease Control and Prevention, National Institute for Occupational Safety and Health, DHHS (NIOSH) Publication 2018–130, https://doi.org/10.26616/NIOSHPUB2018130

[10] van den Dobbelsteen JJ, van Straten BJ, Horeman T. A Comparison of Particle Filter Efficiency Measurements for Protective Masks using Particle Counters with Different Flow Rates. TU-Delft repository 2020.

[11] van Straten B, de Man P, van den Dobbelsteen J, Koeleman H, van der Eijk A, Horeman T. Sterilization of disposable face masks by means of standardized dry and steam sterilization processes; an alternative in the fight against mask shortages due to COVID-19. *Journal of Hospital Infection* 2020; epub

[12] RIVM, 2020 March 18. https://www.rivm.nl/en/documenten/reuse-of-ffp2-masks

[13] Lin T-H, Chen C-C, Huang S-H, Kuo C-W, Lai C-Y, Lin W-Y. Filter quality of electret masks in filtering 14.6–594 nm aerosol particles: Effects of five decontamination methods. PLoS ONE 12(10): e0186217

For peer review only

[14] Noti JD, Lindsley WG, Blachere FM, Cao G, Kashon ML, Thewlis RE, et al. Detection of infectious influenza virus in cough aerosols generated in a simulated patient examination room. Clin Infect Dis 2012;54:1569-77.

[15] Lewis D. Is the coronavirus airborne? Experts can't agree. Nature 2020;580:175.

Contributors

REH, BvS, BvM, JB, TH contributed to the study design and acquisition of data. REH, BvS and TH analyzed and interpreted the data. JB, JJvdD, JRMvdS contirbuted to the interpretation. REH drafted the initial manuscript and all authors critically revised the manuscript and gave final approval.

Acknowledgements

The authors would like to thank Jan-Willem Klok and Tomas Lenssen for testing of all samples on the test setup.

Funding statement

This work of research received no specific grant from any funding agency in the public, commercial or not-for-profit sectors.

Competing interests

None declared.

Patient consent for publication

Not required.

Ethics approval:

Not required.

Data availability statement:

All data will be made available on https://repository.tudelft.nl/

For peer review only

FIGURE LEGENDS

Figure 1. Panel A (left side) illustrates the measurement setup used to measure the particle penetration capacity of different respirators. The particle chamber is connected to the Lighthouse 3100 with a custom connecter and 5 mm tube. An adjustable removable transparent lid is used to press the filter material of a mask airtight on the rim of the open particle chamber for accurate measurements. Panel B (right side) shows an expansion of the setup with an additional pressure sensor and pressure-to-voltage converter.

Figure 2. Filter quality of autoclave-decontaminated respirators (retainment of 0.3µm particles) of unused, and one-time, two-time, and three-time autoclave sterilization

For peer review only

Table 1. Filter capacity of the tested respirators by particle size

Respirator	Condition (new/sterilized)	No of samples	0.3 µm	0.5 µm	1.0 µm	5.0 µm
3M Aura 1862+	New	4	99.3±0.3	99.7±0.0	99.8±0.1	99.9±0.2
	1x	4	97.0±0.8	99.0±0.0	99.0±0.5	100±0.0
	2x	8	94.2±1.3	97.4±0.5	98.9±0.3	99.9±0.1
	3x	4	94.4±1.3	97.5±0.9	98.8±0.4	100±0.0
3M Aura 9322+	New	2	96.7±0.2	99.1±0.0	99.7±0.0	99.3±0.3
	1x	2	91.0±1.0	99.0±0.0	100±0.0	100±0.0
	2x	2	77.5±1.5	85.5±0.5	89.5±0.5	98.0±1.0
San Huei 2920V	New	2	95.5±0.5	99.0±0.0	100±0.0	100±0.0
	1x	8	92.3±1.6	97.8±0.7	99.1±0.3	96.0±5.4

For peer review only

	2x	6	90.2±0.7	96.8±0.35	99.0±0.0	97.8±2.3
Maco Pharma ZZM002	1x	8	89.3±3.6	96.8±1.2	98.9±0.3	99.8±0.4
	2x	6	90.2±0.7	96.8±0.35	99.0±0.0	97.8±2.3
Safe Worker 1016	New	2	96.5±0.5	98.0±0.0	60.5±1.5	99±.0.0
	1x	8	60.3±5.3	81.6±4.9	90.1±5.3	91.5±18.4

For peer review only

For peer review only

For peer review only - http://bmjopen.bmj.com/site/about/guidelines.xhtml

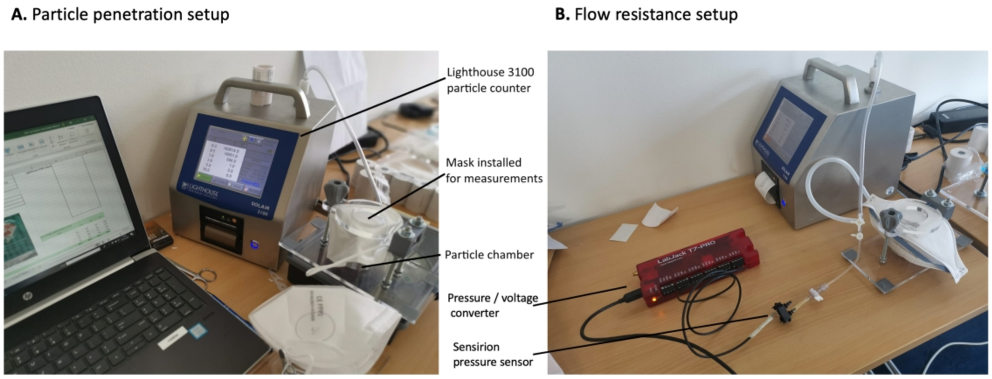
A. Particle penetration setup
B. Flow resistance setup
Lighthouse 3100
particle counter
Mask installed
for measurements
Particle chamber
Pressure / voltage
converter
Sensirion
pressure sensor

Figure 1

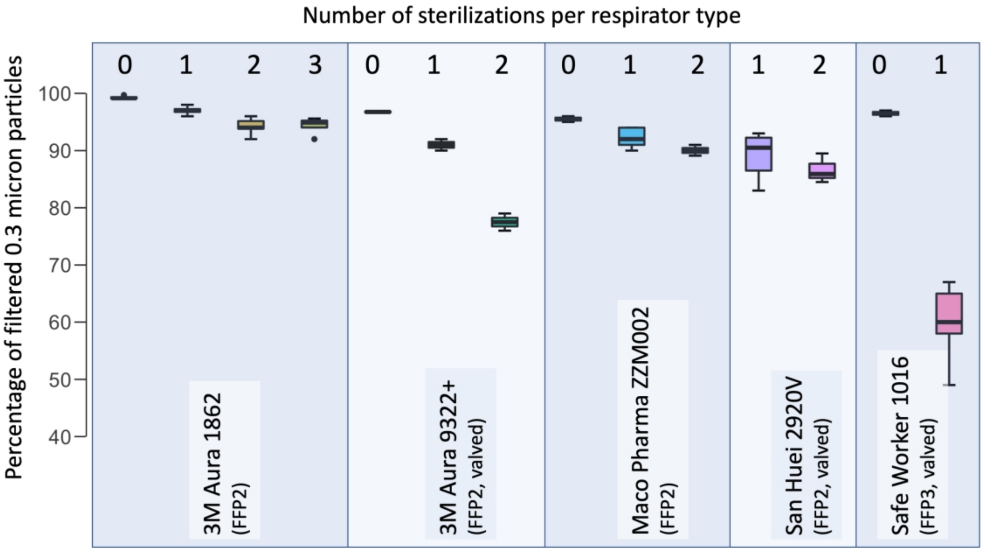
Number of sterilizations per respirator type
0
1
2
3
0
1
2
0
1
2
1
2
0
1
Percentage of filtered 0.3 micron particles
100
90
80
70
60
50
40
3M Aura 1862
(FFP2)
3M Aura 9322+
(FFP2, valved)
Maco Pharma ZZM002
(FFP2)
San Huei 2920V
(FFP2, valved)
Safe Worker 1016
(FFP3, valved)

Figure 2

TIDieR
Template for Intervention Description and Replication

The TIDieR (Template for Intervention Description and Replication) Checklist*:

Information to include when describing an intervention and the location of the information

Item number	Item	Where located ** Primary paper (page or appendix number)	Other † (details)
	**BRIEF NAME**		
**1.**	Provide the name or a phrase that describes the intervention.	1	
	**WHY**		
**2.**	Describe any rationale, theory, or goal of the elements essential to the intervention.	2	
	**WHAT**		
**3.**	Materials: Describe any physical or informational materials used in the intervention, including those provided to participants or used in intervention delivery or in training of intervention providers. Provide information on where the materials can be accessed (e.g. online appendix, URL).	5-7	
**4.**	Procedures: Describe each of the procedures, activities, and/or processes used in the intervention, including any enabling or support activities.	5-7	
	**WHO PROVIDED**		
**5.**	For each category of intervention provider (e.g. psychologist, nursing assistant), describe their expertise, background and any specific training given.	5-7	
	**HOW**		
**6.**	Describe the modes of delivery (e.g. face-to-face or by some other mechanism, such as internet or telephone) of the intervention and whether it was provided individually or in a group.	5-7	
	**WHERE**		
**7.**	Describe the type(s) of location(s) where the intervention occurred, including any necessary infrastructure or relevant features.	5-7	

For peer review only

For peer review only - http://bmjopen.bmj.com/site/about/guidelines.xhtml

	**WHEN and HOW MUCH**		
**8.**	Describe the number of times the intervention was delivered and over what period of time including the number of sessions, their schedule, and their duration, intensity or dose.	______5-8_____	______________
	**TAILORING**		
**9.**	If the intervention was planned to be personalised, titrated or adapted, then describe what, why, when, and how.	______5-8_____	______________
	**MODIFICATIONS**		
**10.‡**	If the intervention was modified during the course of the study, describe the changes (what, why, when, and how).	_____N/A_____	______________
	**HOW WELL**		
**11.**	Planned: If intervention adherence or fidelity was assessed, describe how and by whom, and if any strategies were used to maintain or improve fidelity, describe them.	_____N/A_____	______________
**12.‡**	Actual: If intervention adherence or fidelity was assessed, describe the extent to which the intervention was delivered as planned.	_____N/A_____	______________

** **Authors** - use N/A if an item is not applicable for the intervention being described. **Reviewers** – use '?' if information about the element is not reported/not sufficiently reported.

† If the information is not provided in the primary paper, give details of where this information is available. This may include locations such as a published protocol or other published papers (provide citation details) or a website (provide the URL).

‡ If completing the TIDieR checklist for a protocol, these items are not relevant to the protocol and cannot be described until the study is complete.

* We strongly recommend using this checklist in conjunction with the TIDieR guide (see *BMJ* 2014;348:g1687) which contains an explanation and elaboration for each item.

* The focus of TIDieR is on reporting details of the intervention elements (and where relevant, comparison elements) of a study. Other elements and methodological features of studies are covered by other reporting statements and checklists and have not been duplicated as part of the TIDieR checklist. When a **randomised trial** is being reported, the TIDieR checklist should be used in conjunction with the CONSORT statement (see www.consort-statement.org) as an extension of **Item 5 of the CONSORT 2010 Statement.** When a **clinical trial protocol** is being reported, the TIDieR checklist should be used in conjunction with the SPIRIT statement as an extension of **Item 11 of the SPIRIT 2013 Statement** (see www.spirit-statement.org). For alternate study designs, TIDieR can be used in conjunction with the appropriate checklist for that study design (see www.equator-network.org).

For peer review only - http://bmjopen.bmj.com/site/about/guidelines.xhtml

For peer review only

BMJ Open

Reprocessing filtering facepiece respirators in primary care using medical autoclave: a prospective, bench-to-bedside, single-center study

Journal:	*BMJ Open*
Manuscript ID	bmjopen-2020-039454.R1
Article Type:	Original research
Date Submitted by the Author:	22-Jun-2020
Complete List of Authors:	Harskamp, Ralf; Amsterdam UMC - Locatie AMC, General Practice<br>van Straten, Bart; Delft University of Technology; Van Straten Medical<br>Bouman, Jonathan; Amsterdam UMC - Locatie AMC<br>van Maltha - van Santvoort, Bernadette; Holendrecht Medical Center<br>van den Dobbelsteen, John; Delft University of Technology<br>van der Sijp, Joost; Medical Centre Haaglanden; GreenCycl<br>Horeman, Tim; Delft University of Technology
**Primary Subject Heading**:	General practice / Family practice
Secondary Subject Heading:	Public health, Respiratory medicine, Infectious diseases, Health services research, Evidence based practice
Keywords:	PRIMARY CARE, Respiratory infections < THORACIC MEDICINE, Public health < INFECTIOUS DISEASES

SCHOLARONE™
Manuscripts

I, the Submitting Author has the right to grant and does grant on behalf of all authors of the Work (as defined in the below author licence), an exclusive licence and/or a non-exclusive licence for contributions from authors who are: i) UK Crown employees; ii) where BMJ has agreed a CC-BY licence shall apply, and/or iii) in accordance with the terms applicable for US Federal Government officers or employees acting as part of their official duties; on a worldwide, perpetual, irrevocable, royalty-free basis to BMJ Publishing Group Ltd ("BMJ") its licensees and where the relevant Journal is co-owned by BMJ to the co-owners of the Journal, to publish the Work in this journal and any other BMJ products and to exploit all rights, as set out in our licence.

The Submitting Author accepts and understands that any supply made under these terms is made by BMJ to the Submitting Author unless you are acting as an employee on behalf of your employer or a postgraduate student of an affiliated institution which is paying any applicable article publishing charge ("APC") for Open Access articles. Where the Submitting Author wishes to make the Work available on an Open Access basis (and intends to pay the relevant APC), the terms of reuse of such Open Access shall be governed by a Creative Commons licence – details of these licences and which Creative Commons licence will apply to this Work are set out in our licence referred to above.

Other than as permitted in any relevant BMJ Author's Self Archiving Policies, I confirm this Work has not been accepted for publication elsewhere, is not being considered for publication elsewhere and does not duplicate material already published. I confirm all authors consent to publication of this Work and authorise the granting of this licence.

For peer review only

Reprocessing filtering facepiece respirators in primary care using medical autoclave: a prospective, bench-to-bedside, single-center study

Ralf E. Harskamp [1,2]*, Bart van Straten [3,4]*, Jonathan Bouman [1,2], Bernadette van Maltha – van Santvoort [2], John J. van den Dobbelsteen [3], Joost R.M. van der Sijp [5,6], Tim Horeman [3]

[1] Department of General Practice, Amsterdam UMC – location AMC, Amsterdam, the Netherlands
[2] Holendrecht Medical Center, Amsterdam, the Netherlands
[3] Department of BioMedical Engineering, Faculty of Engineering, Delft University of Technology, Delft , the Netherlands
[4] Van Straten Medical, De Meern, the Netherlands
[5] GreenCycl, De Meern, the Netherlands
[6] Haaglanden Medical Center, The Hague, the Netherlands

* both authors contributed equally to this study

Author for correspondence:
Name: Dr Ralf E. Harskamp, MD, PhD
Role/title: Assistant Professor
Department: Department of General Practice, Division of Primary Care, Public Health and Methodology
Institution: Amsterdam University Medical Centers – Location Academic Medical Center
Address: Room: J2-126, Meibergdreef 9, 1105 AZ, Postbox 22660, 1100 DD Amsterdam, The Netherlands.
Phone: +31(0)20-566.7457
Fax: +31(0)20-566.9186
E-mail for correspondence: r.e.harskamp@gmail.com
Alternative e-mail address : r.e.harskamp@amsterdamumc.nl

For peer review only

Abstract:

Objective

There are widespread shortages of personal protective equipment as a result of the coronavirus disease 2019 (COVID-19) pandemic. Reprocessing filtering facepiece particles (FFP)-type respirators may provide an alternative solution in keeping health care professionals safe.

Design: prospective, bench-to-bedside

Setting: A primary care-based study using FFP-type 2 respirators without exhalation valve (3M Aura 1862+ (20 samples) , Maco Pharma ZZM002 (14 samples)), FFP2 respirators with valve (3M Aura 9322+ (6 samples) and San Huei 2920V (16 samples)), and valved FFP type 3 respirators (Safe Worker 1016 (10 samples)).

Interventions: All masks were reprocessed using a medical autoclave (17 minutes at 121°C with 34-minute total cycle time) and subsequently tested up to 3 times whether these respirators retained their integrity (seal check, pressure drop) and ability to filter small particles (0.3-5.0μm) in the laboratory using a particle penetration test.

Results

We tested 33 respirators, and 66 samples for filter capacity. All 28 FFP-2 respirators retained their shape, whereas half of the decontaminated FFP-3 respirators showed deformities and failed the seal check. The filtering capacity of the 3M Aura 1862 was best retained after 1, 2, and 3 decontamination cycles (0.3μm: 99.3±0.3% (new) versus 97.0±1.3, 94.2±1.3% or 94.4±1.6, p<0.001). Of the other FFP-2 respirators, the San Huei 2920V had 95.5±0.7% at baseline versus 92.3±1.7% versus 90.0±0.7 after one- and two-time decontamination, respectively (p<0.001). The tested FFP-3 respirator (Safe Worker 1016) had a filter capacity of 96.5±0.7% at baseline and 60.3±5.7% after one-time decontamination (p<0.001). Breathing and pressure resistance tests indicated no relevant pressure changes between respirators that were used once, twice or thrice.

Conclusion

This small single-center study shows that selected FFP2-type respirators may be reprocessed for use in primary care, as the tested masks retain their shape, ability to retain particles and breathing comfort after decontamination using a medical autoclave.

For peer review only - http://bmjopen.bmj.com/site/about/guidelines.xhtml

Strengths and limitations of this study

- Pragmatic use of autoclave to decontaminate and reuse filter facepiece respirators

- Combining clinical and laboratory findings to evaluate the safety in terms of shape, ability to retain particles and breathing comfort

- The study is limited in sample size and restricted to selected FFP-2 and FFP-3 respirators

- The study is a first of its kind in primary care settings and thus non-validated

- The study does not provide "hard" clinical evidence in terms of a randomized trial (i.e. reprocessed mask versus usual care)

For peer review only

Introduction

General practitioners (GP) are often the first to evaluate patients with (suspected) coronavirus disease 2019 (COVID-19). This is particularly true in countries where GPs have a gatekeeping role. Given the risk of person-to-person spread this necessitates the need to wear personal protective equipment. [1,2] Unfortunately, most health care facilities are running dangerously low on this equipment. [1,2] In the United States, these critical shortages have resulted in downgrading from respirators to surgical masks and sometimes even resort to home-made cloth-masks. [1] Access to adequate supplies is crucial to preventing transmission of pathogens, especially in resource-limited settings. [3] Reports across several countries found that healthcare workers are more at risk of catching SARS-CoV2 as well as at higher risk of severe COVID-19, possibly due to exposure to higher viral load. [4] The outbreak of COVID-19 in Italy showed that inadequate access to protective equipment is one of the reasons why healthcare workers, and particularly GPs, experienced high rates of infection. [2] Aside from the direct health effects, absenteeism from illness may also negatively affect the health system's capacity to adequately respond to the COVID-19 pandemic. Moreover, it makes healthcare workers feel unsafe and unprotected, which undermines morale as shown in a report on England's National Health Service health workers experiences. [5]

One of the possible (short-term pragmatic) solutions could be the reuse of equipment, and in particular that of respirators. To reuse a mask or respirator, it should be decontaminated first. The method applied should: 1) kill the SARS-CoV-2 virus (diminish the viral load); 2) keep the mask's protective properties (largely) intact, in terms of filter and fit. In primary care, the medical autoclave is normally used to decontaminate surgical instruments. The process of pressurized moist heat destroys microorganisms by the irreversible coagulation and denaturation of enzymes and structural proteins, and has been shown to be effective in respirators contaminated with other viruses, such as H1N1 influenza virus. [2, 6, 7] However, the question is whether the respirator's protective properties in terms of filter function and fit will remain intact when exposing the respirator to steam. We therefore set out to study whether the process of steam sterilization negatively affects the protective properties of commonly used respirators which are designed to protect the wearer against the inhalation of both droplets and particles suspended in the air.

Methods

We reported our findings according to the STROBE checklist and guide (supplemental file), as well as the general principles of reporting a study using the directions provided by the journal. [8]

Study design and setting

The study involved the evaluation of available filtering respirators used to evaluate suspected COVID-19 patients in the Holendrecht Medical Center, in Amsterdam, the Netherlands. For high-risk patients, the center provides GPs with filtering facepiece particles (FFP) type 2 or type 3 for personal protection. For the current study, worn respirators were used for reprocessing using a medical autoclave. After the autoclave procedure the respirators were visually inspected for deformity by two clinical investigators, followed by a seal check. The masks were subsequently marked and sent by courier to the GreenCycl testing laboratory in Utrecht, the Netherlands. At this

For peer review only - http://bmjopen.bmj.com/site/about/guidelines.xhtml

facility, the decontaminated respirators were tested by two laboratory scientists for their filter capacity. For comparison, the results ware compared with the filter capacity of unused, brand-new respirators that were used as a reference. Moreover, a pressure drop test was performed to evaluate whether the breathing resistance altered by the process of decontamination.

Decontamination process

The masks were decontaminated for multiple cycles using a cylindrical chamber tabletop autoclave (Kronos S18, release: E.5.47a, Newmed, Quattro Castella, Italy). This type of vacuum autoclave is typically designed for general practitioner and dental practices and has pre-programmed cycles. The size as well as the cycle times differ from autoclaves typically used in hospitals, which are larger and have longer cycle times, however with comparable peak times regarding decontamination. The Kronos S18 autoclave holds a capacity of 18L or 4 respirators. The autoclave has specific programs for "solid made of rubber and delicate solids", which includes respirators. The sterilization program we used involved a 34-minute cycle, of which the first 12 minutes of the cycle involved preheating, followed by 17 minutes steam decontamination at a temperature of 121 degrees of Celsius, and finished with a 5-minute drying process.

Visual inspection, breathing resistance, and user seal check

After decontamination the respirators were checked for visual deformities of the mask as well as the elastic straps. Subsequently, the respirators were put on to evaluate whether breathing felt normal, followed by the performance of a user seal check. A negative pressure user seal check was used for all respirators in which the clinical investigator inhaled sharply while blocking the paths for air to enter the facepiece. A successful check is when the facepiece collapsed slightly under the negative pressure that was created with this maneuver. For respirators without an exhalation valve the investigator also performed a positive pressure check by exhaling gently while blocking the paths for air to exit the facepiece. A successful check is when the facepiece was slightly pressurized before increased pressure causes outward leakage. [9]

Particle penetration test

At the testing laboratory two independent researchers from the Delft University of Technology tested the masks using a dry particle penetration test setup (***Figure 1***). [10] The equipment involved a SOLAIR 3100 particle counter (Lighthouse Worldwide Solutions Inc, Fremont, CA). The particles are counted within the machine via a tube that is connected to a particle chamber to which the respirator is secured. The transparent lid presses the mask such that it prevents material buckling and creates an airtight seal that only allows air to pass through the material. Before each measurement, a benchmark test is conducted with 28 Liters of surrounding air that is sucked through the particle chamber into the particle counter (Figure 1, top half). The particle counter measures the particles that are naturally present in the air. During benchmark testing no mask is installed. During the test measurement, a mask is installed on the particle chamber (Figure 1, bottom half). Therefore, the 28 liter of surrounding air is sucked through the filter material of the mask and the remaining particles are counted in the categories of 0.3, 0.5, 1.0 and 5.0 microns. The measurements are compared and the filtering capacity derived based on the difference in the readings compared to the benchmark test. A lower number of particles counted after filtering in relation to the benchmark test would indicate better filtering performance. [11] The system setup is

For peer review only - http://bmjopen.bmj.com/site/about/guidelines.xhtml

more conservative than the NEN-149 standard, which means the resulting filter capacity percentages cannot be translated directly to the known FFP1, 2 and 3 standards. However, as the filter capacity of a new mask is known, the measurement results do show the remaining filter capacity and therefore indicates whether a mask type deteriorates after steam decontamination.

Flow resistance

The setup was expanded with an additional pressure sensor (SDP 816-500Pa Sensirion #1230319) and flow adjustment valve in order to investigate whether the pressure delta over the mask material changed after decontamination cycles. The Sensirion pressure sensor was connected with a T-piece between the particle chamber and Lighthouse 3100. An additional valve was used to adjust the input pressure within range of the sensor. A LabJack T7 analogue input device was used to convert the output from the pressure sensor to an output voltage of 0 to 5 Volt. An output value of 5V was representing 500Pa and set as 100% of input pressure. the atmospheric input pressure of 2.42 Volt was defined as 0% output. Measurements were conducted with a constant air speed of 20.7 meter per seconds at the opening of the particle chamber.

Outcomes of interest

The outcomes of interest involved: 1) signs of deformity of the respirator, which was performed by visual inspection; and 2) the percentage of filtered particles with a diameter of 0.3 µm. This diameter is clinically relevant, given that to meet the FFP-2 standards a mask should filter 94% of all 0.3 µm particles, whereas 99% of these particles should be filtered to meet the FFP-3 standard.

Statistical analysis

The study involved descriptive analyses, with numbers and percentages, and comparisons were performed using an alpha of 0.05 for statistical significance. The findings of the filter tests are visually displayed in Box plots, and presented as mean and standard deviation. We used JASP statistical software (version 0.10.2, University of Amsterdam, the Netherlands).

Patient and Public Involvement:

Patients and/or the public were not involved in the design, or conduct, or reporting, or dissemination plans of our research.

Results

We obtained 33 respirators, of which 28 were used during consultation or high-risk home visits of COVID-19 suspected patients at the Holendrecht Medical Center in March/April 2020. The facemasks were FFP-2 respirators (3M Aura 1862+, Maco Pharma ZZM002), FFP2 respirators with exhalation valve (3M Aura 9322+ and San Huei 2920V), or FFP3 respirators (Safe Worker 1016). The 28 used respirators (including 4 FFP3 respirators) underwent decontamination, with the remaining 5 serving as a reference (as they did not to undergo decontamination).

Visual inspection, breathing resistance, and user seal check

After the decontamination process all FFP-2 respirators retained their shape and were without visible damage. When fitting, the elastic bands of all masks still functioned normally, with no difference from non-decontaminated masks in terms of breathing

For peer review only - http://bmjopen.bmj.com/site/about/guidelines.xhtml

resistance. The seal checks also did not reveal significant air leakage suggesting poor fit. However, unlike the FFP-2 respirators, two out of the four FFP3 respirators (50%) showed signs of deformation, with a crumbled appearance, and failed seal check test.

Filter capacity of decontaminated respirators

For the particle penetration test, a total of 66 samples were tested from 33 respirators. The results of the filter capacity for 0.3 microns are illustrated in ***Figure 2*** and for larger particles are displayed in ***Table 1.*** Of the tested FFP-2 respirators we found that the 3M Aura 1862+ remained close to its original filtering capacity after one-, two-time, and three-time decontamination (0.3μm: 99.3±0.3% versus 97.0±1.3, 94.2±1.3% or 94.4±1.6, respectively, p<0.001). The 3M Aura 9322+ (with valve) had a filter capacity of 96.8±0.2% without decontamination versus 91.0±1.4% and 77.5±2.1% after one- or two-time decontamination (p<0.001). The Maco Pharma ZZM002 FFP-2 mask did not have a reference mask, but after one- and two-time decontamination the filter capacities were 89.3±3.9% and 86.6±2.6%, respectively. The San Huei 2920V respirator had 95.5±0.7% at baseline versus 92.3±1.7% versus 90.0±0.7 after one- and two-time decontamination (p<0.001). Finally, the tested FFP-3 respirator (Safe Worker 1016) had a filter capacity of 96.5±0.7% at baseline and 60.3±5.7% after one-time decontamination (p<0.001).

Flow resistance

For the breathing resistance test we tested 6 FFP-2 respirators (3M Aura 1862+): two were used once and reprocessed, two were used twice and reprocessed after each use and two were used for 3 times and reprocessed after each use. The average pressure did not increase with the number of reuses (35.6±0.3%, 35.4±0.0%, 36.7±0.3%, respectively)

Discussion

The COVID-19 pandemic has caused major shortages of PPE, including protective respirators. While production has increased, shortages are so high that reprocessing of used respirators and respirators is probably one of the only viable short-term solutions. In primary care, a tabletop autoclave would be a pragmatic choice, as the device is readily available in practices for decontamination of surgical and gynecological instruments. In this study we found that steam decontamination at 121 degrees Celsius may provide a viable option for selected respirators, but it also sheds a light on the variability in the protective properties of the various available respirators and respirators. Of the tested respirators, the filter capacity of the 3M Aura 1862+ respirator fared best with a consistently high filter capacity for the 0.3 μm particle size category and above after multiple cycles of steam decontamination. Moreover, there are no indications that the respirator becomes harder to breathe through and thus more uncomfortable to wear. We also observed that with multiple decontamination cycles , the mean particle filtration efficiency for 5 microns becomes slightly lower than for 1 micron particles for some of the respirators. We speculate that perhaps larger 5 micron particles are more likely to remain trapped in the filter material after use and during reprocessing and are subsequently sucked out of the material during testing, which in turn negatively affect the filter readings.

Findings in relationship to FFP-2 and FFP-3 standards

For peer review only - http://bmjopen.bmj.com/site/about/guidelines.xhtml

The particle chamber used in this study appears to be more stringent (more sensitive) that the NEN-149 criteria that are used for FFP-2 and FFP-3 norms. We performed a cross-check with 4 KN95 respirators which showed that measurements of 67% and 82% particle retention at 0.3 and 0.5 microns on average, using our setup still resulted in approval for use according to the FFP-2 norm when measured according to the NEN-149, based on a continuous flow setup (Kalibra, Delft, the Netherlands). Therefore, apart from the Safe Worker 1016, all other mask types will likely still comply with NEN-149 FFP-2 threshold values.

Study limitations

Our study involved a pragmatic study with a limited sample size of respirators and respirators available in our practice. Our study did not involve testing of surgical masks or FFP-1 masks, and we do not know whether reprocessing of these materials would still provide adequate protection based on their respective standards. Furthermore, we did not perform a laboratory-based Fit test. Also, we presumed that solid particles of 0.3-5 microns are of relevance and behave similar as droplets that normally carry viruses from one person to another. Smaller particles of 0.1-0.2 microns were not included in this study, as we deemed these to contribute less to the spread of the virus. However, this is an assumption as we do not yet know for certain at what particle size viral transmission is still possible and respirators provide adequate protection. [12] Although the used flow rate of 28 liter/min is in the range of the normal breathing conditions it did not fully comply with the requirement for the EN-149 sampling flow rate. Therefore, additional studies should also include the influence on flow rate on particle filtration capacity.

Prior studies on heat as a decontamination method

From the literature there is a consensus that thermal inactivation is a very efficient technique to eliminate viruses. [2,6,7,11,13] Prior research indicates that steam decontamination for a total of 5 minutes is sufficient to completely inactivate the avian coronavirus, for instance [14]. Moreover, thermal inactivation of viruses, such as SARS-CoV, porcine and avian coronaviruses, poliovirus, and influenza virus do not appear to differ much. [14-16] For SARS-CoV-2, a study by Fisher *et al* studied inactivation of this particular virus using four modalities, including dry heat (70 degrees Celsius). The study found that dry heat kills SARS-CoV-2 at similar speed to UV. [17] Based on these combined data it is assumed that decontamination via autoclave is also sufficient to inactivate SARS-CoV-2.

Prior studies on the reuse of respirators

One possible concern with respirators is that extended use and reuse could reduce its protective effectiveness in terms of filter function and fit. Lin *et al* assessed the impact of steam decontamination and other decontamination procedures on the filter capacity of respirators. [18] In this study, the authors found that one of the decontamination processes that appeared effective for N95 respirators was the medical autoclave, in which they exposed the respirators to saturated steam at 121 degrees Celsius for 15 minutes. They found that filter quality (≥95%) of the masks remained intact using a range of particles. These findings are comparable to those we present in this paper. Besides filter capacity, the integrity of facepiece respirators should also be kept in mind. When exposing masks to higher temperatures (132-140 centigrade), respirators may become deformed, as was shown in a recent study of the Dutch Centers of Disease

For peer review only - http://bmjopen.bmj.com/site/about/guidelines.xhtml

Control (RIVM) [19]. In our study, we did not find such deformity at a lower temperature.

Implications for practice

For COVID-19 like for other viruses, transmission can occur via droplets or aerosols. [1-4] Thus personal protection is warranted to avoid catching COVID-19. Currently there is no evidence on which type of face mask offers best protection for COVID-19. Prior studies with influenza viral particles showed that FFP2 respirators may provide better protection that surgical masks, when used appropriately. [20] In this COVID-19 pandemic, it is thought that the use of surgical masks may be sufficient for consultations with only limited person-to-person exposure. However, it is much less certain whether surgical masks will provide adequate protection during longer consultations or back-to-back consultations with patients with suspected COVID-19 in a closed consultation room. [21] In these instances, respirators may be preferable. Given, the limited availability, reusing FFP-2 type respirators may provide a second-best alternative that can be readily performed in primary care and other low-resource settings using a table-top medical autoclave, as described in this study. In the unlikely event of performing or present for an aerosol-generating procedure, the CDC states that reprocessed respirators should not be used [1]. Please also be advised about the following: the use of exhalation valve-type respirators for healthcare workers is debatable. The use of an exhalation valve does not appear to offer a benefit in physiological burden over a respirator without valve for the wearer [22], whilst it exposes (the often vulnerable) patient to the user's exhalation breath. As such, when available a respirator without a exhalation valve should be preferred. We would also advice to mark reprocessed respirators with the wearer's initials as well as the number of cycles. Finally, physicians should familiarize themselves on how to perform a user seal check. This procedure should be performed every time a respirator is put on, and assures that the respirator is being properly worn. Details on how to perform this simple check can be found at the website of the CDC. [9]

Conclusion

This study shows that selected FFP2 respirators may be reprocessed for use in primary care, as the respirators retain their shape, ability to retain particles and breathing comfort after decontamination using a medical autoclave. However, future studies are warranted to confirm our findings.

References

[1] https://www.cdc.gov/coronavirus/2019-nCoV/hcp/index.html

[2] Ranney ML, Griffeth V, Jha AK. Critical supply shortages - the need for ventilators and personal protective equipment during the Covid-19 pandemic. N Engl J Med 2020; March 25th, https://www.nejm.org/doi/full/10.1056/NEJMp2006141

[3] Honda H, Iwata K. Personal protective equipment and improving compliance among healthcare workers in high-risk settings. Curr Opin Infect Dis 2016;29:400-6

For peer review only - http://bmjopen.bmj.com/site/about/guidelines.xhtml

[4] Heneghan C, Brassey J, Jefferson T. SARS-CoV-2 viral load and the severity of COVID-19. https://www.cebm.net/covid-19/sars-cov-2-viral-load-and-the-severity-of-covid-19/

[5] Horton R. Offline: COVID-19 and the NHS – "a national scandal". The Lancet 2020;395:P01229.

[6] Rutala WA, Weber DJ. Sterilization in healthcare facilities, 2008. Update: May 2019. https://www.cdc.gov/infectioncontrol/guidelines/disinfection/

[7] Heimbuch BK, Wallace WH, Kinney K, Lumey AE, Wu C-Y, Woo M-H, Wander JD. A pandemic influence preparedness study: use of energetic methods to decontaminate filtering facepiece respirators contaminated with H1N1 aerosols and droplets. Am J Infect Control 2011;39:e1-9.

[8] von Elm E, Altman DG, Egger M, et al. Strengthening the Reporting of Observational Studies in Epidemiology (STROBE)statement: guidelines for reporting observational studies.. BMJ 2007; 335:806-8.

[9] NIOSH. Filtering out Confusion: Frequently Asked Questions about Respiratory Protection, User Seal Check. By Krah J., Shamblin M., and Shaffer R. Pittsburgh, PA: U.S. Department of Health and Human Services, Centers for Disease Control and Prevention, National Institute for Occupational Safety and Health, DHHS (NIOSH) Publication 2018–130, https://doi.org/10.26616/NIOSHPUB2018130

[10] van den Dobbelsteen JJ, van Straten BJ, Horeman T. A Comparison of Particle Filter Efficiency Measurements for Protective Masks using Particle Counters with Different Flow Rates. TU-Delft repository 2020.

[11] van Straten B, de Man P, van den Dobbelsteen J, Koeleman H, van der Eijk A, Horeman T. Sterilization of disposable face masks by means of standardized dry and steam sterilization processes; an alternative in the fight against mask shortages due to COVID-19. *Journal of Hospital Infection* 2020; epub

[12] Jayaweera M, Perera H, Gunawardana B, Manatunge J. Transmission of COVID_19 virus by droplets and aerosols: a critical review on the unresolved dichotomy. Environ Res 2020; epub Jun 13; doi: 10.1016/j.envres.2020.109819

[13] Pagat A-M, Seux-Goepfert R, Lutsch C, et al. Evaluation of SARS-coronavirus decontamination procedures. Applied Biosafety 2007;12:100-108.

[14] Ma Q-X, Shan H, Zhang C-M, et al. Decontamination of face masks with steam for mask reuse in fighting the pandemic COVID-19: experimental supports. J of Med Vir 2020; epub. https://doi.org/10.1002/jmv.25921.

[15] Zhang Q, Zhao Q. Inactivating porcine coronavirus before nuclei acid isolation with the temperature higher than 56°C damages its genome integrity seriously. bioRxiv preprint, 2020. https://doi.org/10.1101/2020.02.20.958785.

For peer review only

For peer review only - http://bmjopen.bmj.com/site/about/guidelines.xhtml

[16] Lu H, Castro, AE, Pennick K. et al. Survival of avian influenza virus H7N2 in SPF chickens and their environments. Avian Diseases 2003:47, 1015-1021.

[17] Fisher RJ, Morris DH, van Doremalen N, et al. Assessment of N95 respirator decontamination and re-use for SARS-CoV-2. https://www.medrxiv.org/content/10.1101/2020.04.11.20062018v2.full.pdf

[18] Lin T-H, Chen C-C, Huang S-H, Kuo C-W, Lai C-Y, Lin W-Y. Filter quality of electret masks in filtering 14.6–594 nm aerosol particles: Effects of five decontamination methods. PLoS ONE 2017:12(10): e0186217

[19] RIVM, 2020 March 18. https://www.rivm.nl/en/documenten/reuse-of-ffp2-masks

[20] Noti JD, Lindsley WG, Blachere FM, Cao G, Kashon ML, Thewlis RE, et al. Detection of infectious influenza virus in cough aerosols generated in a simulated patient examination room. Clin Infect Dis 2012;54:1569-77.

[21] Lewis D. Is the coronavirus airborne? Experts can't agree. Nature 2020;580:175.

[22] Roberge RJ, Coca A, Williams WJ, Powell JB, Palmiero AJ. Physiological impact of the N95 filtering facepiece respirator on healthcare workers. Respir Care 2010;55:569-77.

Contributors

REH, BvS, BvM, JB, TH contributed to the study design and acquisition of data. REH, BvS and TH analyzed and interpreted the data. JB, JJvdD, JRMvdS contirbuted to the interpretation. REH drafted the initial manuscript and all authors critically revised the manuscript and gave final approval.

Acknowledgements

The authors would like to thank Jan-Willem Klok and Tomas Lenssen for testing of all samples on the test setup.

Funding statement

This work of research received no specific grant from any funding agency in the public, commercial or not-for-profit sectors.

Competing interests

REH, BvS, JB, TH have nothing to declare. BvS has shares in the company Van Straten Medical and Greencycl that build and owns a part of the measurement systems used for the execution of this study. Greencycle facilitated the Measurements.

Patient consent for publication

Not required.

Ethics approval:

Not required.

For peer review only - http://bmjopen.bmj.com/site/about/guidelines.xhtml

Data availability statement:
All data will be made available on https://repository.tudelft.nl/

For peer review only

FIGURE LEGENDS

Figure 1. illustrates the measurement setup used to measure the particle penetration capacity of different respirators. The particle chamber is connected to the Lighthouse 3100 with a custom connecter and 5 mm tube. An adjustable removable transparent lid is used to press the filter material of a mask airtight on the rim of the open particle chamber for accurate measurements.

Figure 2. Filter quality of autoclave-decontaminated respirators (retainment of 0.3μm particles) of unused, and one-time, two-time, and three-time autoclave sterilization

For peer review only

For peer review only - http://bmjopen.bmj.com/site/about/guidelines.xhtml

Table 1. Filter capacity of the tested respirators by particle size

Respirator	Condition (new/decontaminated)	No of samples	0.3 µm	0.5 µm	1.0 µm	5.0 µm
3M Aura 1862+	New	4	99.3±0.3	99.7±0.0	99.8±0.1	99.9±0.2
	1x	4	97.0±0.8	99.0±0.0	99.0±0.5	100±0.0
	2x	8	94.2±1.3	97.4±0.5	98.9±0.3	99.9±0.1
	3x	4	94.4±1.3	97.5±0.9	98.8±0.4	100±0.0
3M Aura 9322+	New	2	96.7±0.2	99.1±0.0	99.7±0.0	99.3±0.3
	1x	2	91.0±1.0	99.0±0.0	100±0.0	100±0.0
	2x	2	77.5±1.5	85.5±0.5	89.5±0.5	98.0±1.0
San Huei 2920V	New	2	95.5±0.5	99.0±0.0	100±0.0	100±0.0
	1x	8	92.3±1.6	97.8±0.7	99.1±0.3	96.0±5.4

For peer review only

	2x	6	90.2±0.7	96.8±0.35	99.0±0.0	97.8±2.3
Maco Pharma ZZM002	1x	8	89.3±3.6	96.8±1.2	98.9±0.3	99.8±0.4
	2x	6	90.2±0.7	96.8±0.35	99.0±0.0	97.8±2.3
Safe Worker 1016	New	2	96.5±0.5	98.0±0.0	60.5±1.5	99±.0.0
	1x	8	60.3±5.3	81.6±4.9	90.1±5.3	91.5±18.4

For peer review only

For peer review only

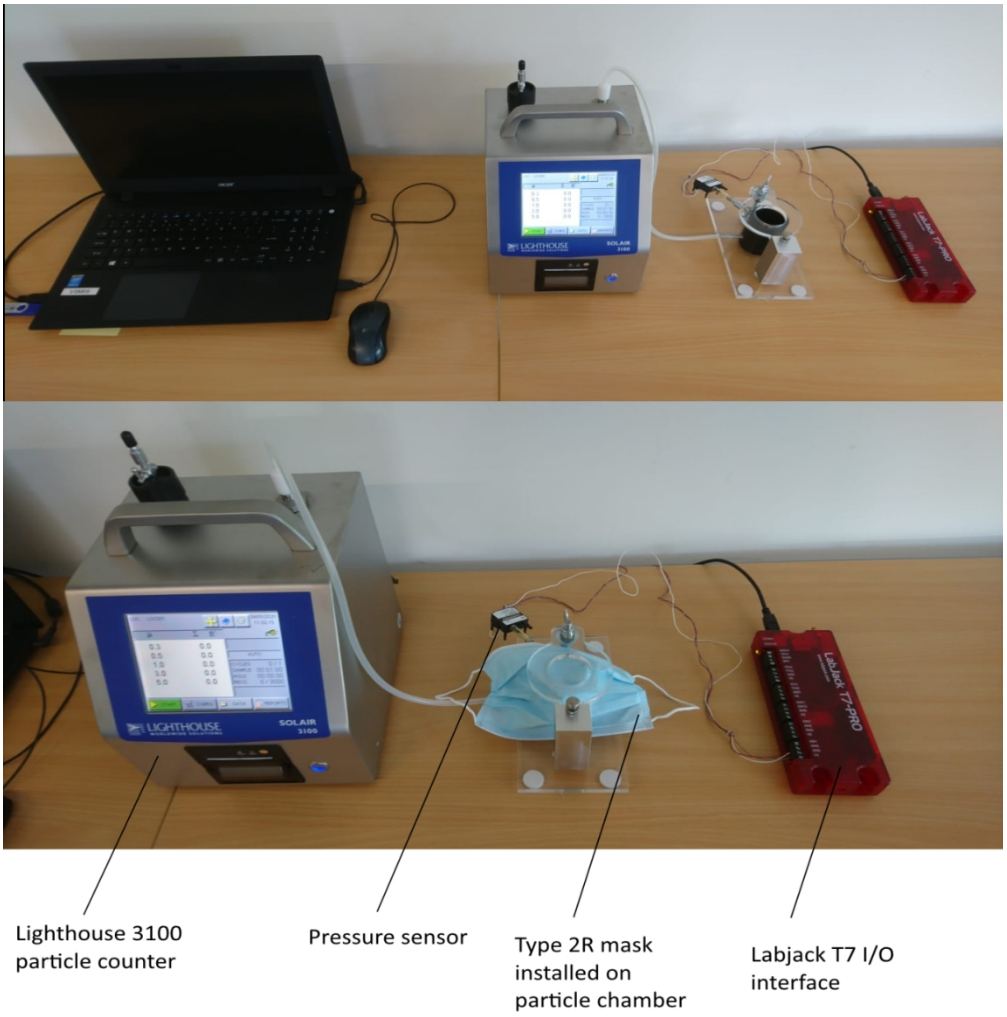

Figure 1

147x150mm (220 x 220 DPI)

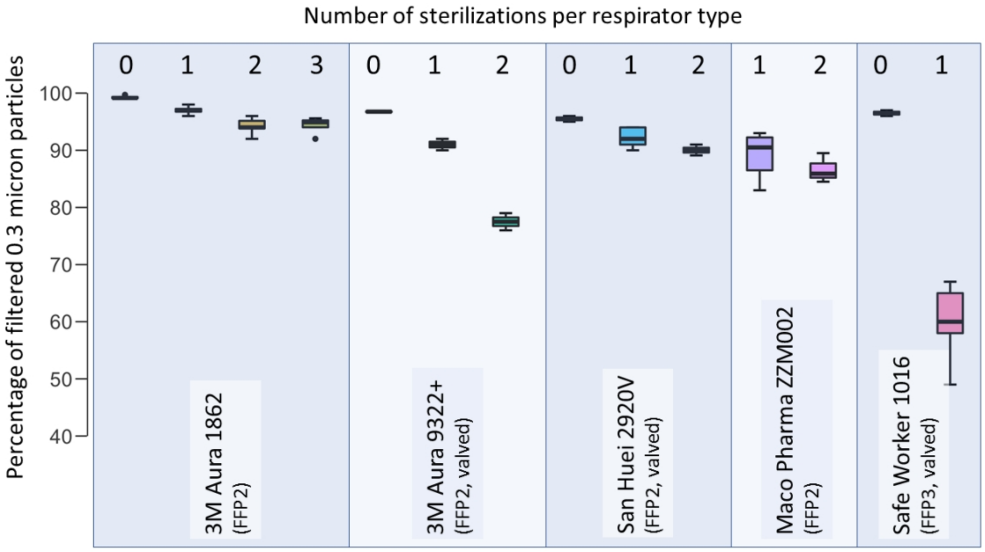

Figure 2

338x190mm (96 x 96 DPI)

STROBE 2007 (v4) checklist of items to be included in reports of observational studies in epidemiology*
Checklist for cohort, case-control, and cross-sectional studies (combined)

Section/Topic	Item #	Recommendation	Reported on page #
**Title and abstract**	1	(*a*) Indicate the study's design with a commonly used term in the title or the abstract	1,2
		(*b*) Provide in the abstract an informative and balanced summary of what was done and what was found	2
**Introduction**			
Background/rationale	2	Explain the scientific background and rationale for the investigation being reported	4
Objectives	3	State specific objectives, including any pre-specified hypotheses	4
**Methods**			
Study design	4	Present key elements of study design early in the paper	4,5
Setting	5	Describe the setting, locations, and relevant dates, including periods of recruitment, exposure, follow-up, and data collection	5
Participants	6	(*a*) *Cohort study*—Give the eligibility criteria, and the sources and methods of selection of participants. Describe methods of follow-up<br>*Case-control study*—Give the eligibility criteria, and the sources and methods of case ascertainment and control selection. Give the rationale for the choice of cases and controls<br>*Cross-sectional study*—Give the eligibility criteria, and the sources and methods of selection of participants	5,6
		(*b*) *Cohort study*—For matched studies, give matching criteria and number of exposed and unexposed<br>*Case-control study*—For matched studies, give matching criteria and the number of controls per case	
Variables	7	Clearly define all outcomes, exposures, predictors, potential confounders, and effect modifiers. Give diagnostic criteria, if applicable	5,6
Data sources/ measurement	8*	For each variable of interest, give sources of data and details of methods of assessment (measurement). Describe comparability of assessment methods if there is more than one group	*5,6*
Bias	9	Describe any efforts to address potential sources of bias	8
Study size	10	Explain how the study size was arrived at	-
Quantitative variables	11	Explain how quantitative variables were handled in the analyses. If applicable, describe which groupings were chosen and why	NA
Statistical methods	12	(*a*) Describe all statistical methods, including those used to control for confounding	6
		(*b*) Describe any methods used to examine subgroups and interactions	NA
		(*c*) Explain how missing data were addressed	NA
		(*d*) *Cohort study*—If applicable, explain how loss to follow-up was addressed<br>*Case-control study*—If applicable, explain how matching of cases and controls was addressed	NA

For peer review only

		Cross-sectional study—If applicable, describe analytical methods taking account of sampling strategy	
		(*e*) Describe any sensitivity analyses	NA
**Results**			
Participants	13*	(a) Report numbers of individuals at each stage of study—eg numbers potentially eligible, examined for eligibility, confirmed eligible, included in the study, completing follow-up, and analysed	6,7
		(b) Give reasons for non-participation at each stage	6,7
		(c) Consider use of a flow diagram	6,7
Descriptive data	14*	(a) Give characteristics of study participants (eg demographic, clinical, social) and information on exposures and potential confounders	6,7
		(b) Indicate number of participants with missing data for each variable of interest	6,7
		(c) *Cohort study*—Summarise follow-up time (eg, average and total amount)	6,7
Outcome data	15*	*Cohort study*—Report numbers of outcome events or summary measures over time	6,7
		Case-control study—Report numbers in each exposure category, or summary measures of exposure	6,7
		Cross-sectional study—Report numbers of outcome events or summary measures	6,7
Main results	16	(*a*) Give unadjusted estimates and, if applicable, confounder-adjusted estimates and their precision (eg, 95% confidence interval). Make clear which confounders were adjusted for and why they were included	6,7
		(*b*) Report category boundaries when continuous variables were categorized	6,7
		(*c*) If relevant, consider translating estimates of relative risk into absolute risk for a meaningful time period	6,7
Other analyses	17	Report other analyses done—eg analyses of subgroups and interactions, and sensitivity analyses	6,7
**Discussion**			
Key results	18	Summarise key results with reference to study objectives	7,8
Limitations	19	Discuss limitations of the study, taking into account sources of potential bias or imprecision. Discuss both direction and magnitude of any potential bias	7,8
Interpretation	20	Give a cautious overall interpretation of results considering objectives, limitations, multiplicity of analyses, results from similar studies, and other relevant evidence	7,8
Generalisability	21	Discuss the generalisability (external validity) of the study results	7,8
**Other information**			
Funding	22	Give the source of funding and the role of the funders for the present study and, if applicable, for the original study on which the present article is based	-

*Give information separately for cases and controls in case-control studies and, if applicable, for exposed and unexposed groups in cohort and cross-sectional studies.
Note: An Explanation and Elaboration article discusses each checklist item and gives methodological background and published examples of transparent reporting. The STROBE checklist is best used in conjunction with this article (freely available on the Web sites of PLoS Medicine at http://www.plosmedicine.org/, Annals of Internal Medicine at http://www.annals.org/, and Epidemiology at http://www.epidem.com/). Information on the STROBE Initiative is available at www.strobe-statement.org.

For peer review only
